# Supplementary material for: Development and Characterization of Membranes with PVA Containing Silver Particles: A Study of the Addition and Stability
Source: Polymers (Basel). 2020 Aug 27;12(9):1937. doi: 10.3390/polym12091937 (PMC7565032; doi:10.3390/polym12091937)
Supplement: Supplementary file 1 [file polymers-12-01937-s001.pdf]

Supplementary Materials

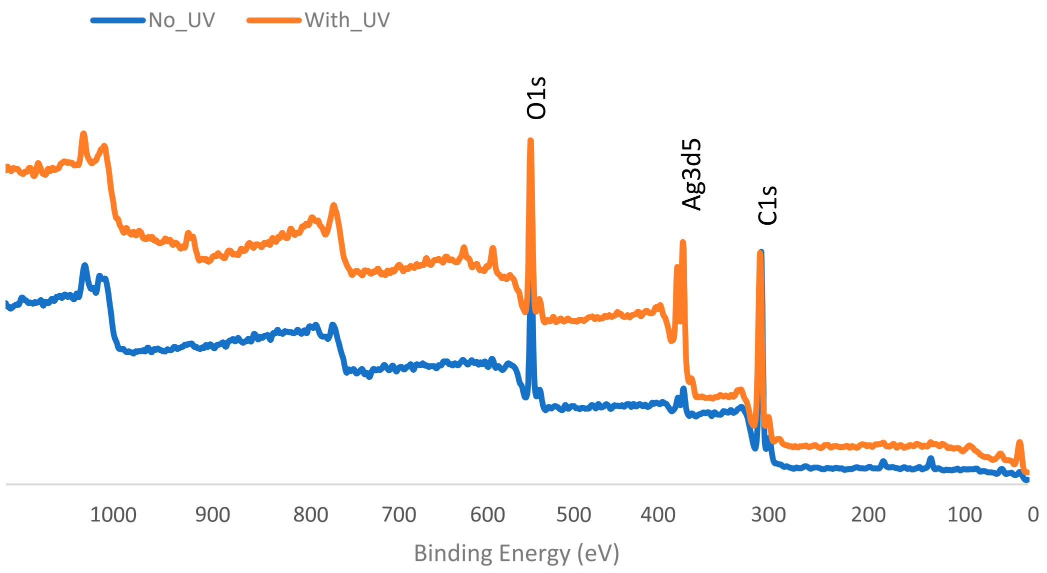

Fig S1. XPS spectra of PVA-Ag and PVA-UV of spectral region 0-1000 eV.

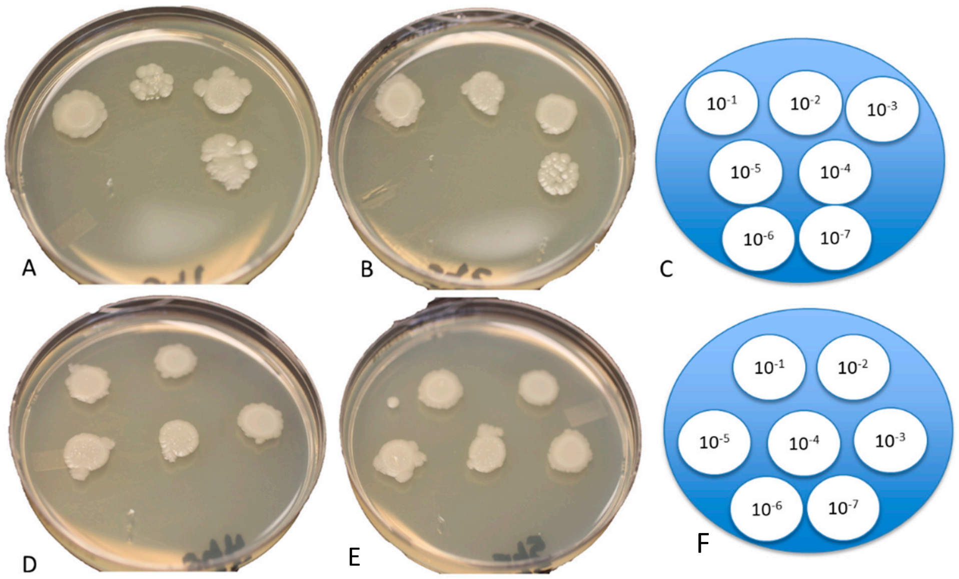

Fig S2. Bacteria plating following incubation with membrane embedded with silver (A) 1 hour post exposure, (B) 2 hours post exposure, (D) 4 hours post exposure and (E) 5 hours exposure, and maps of dilution colonies (C and F).

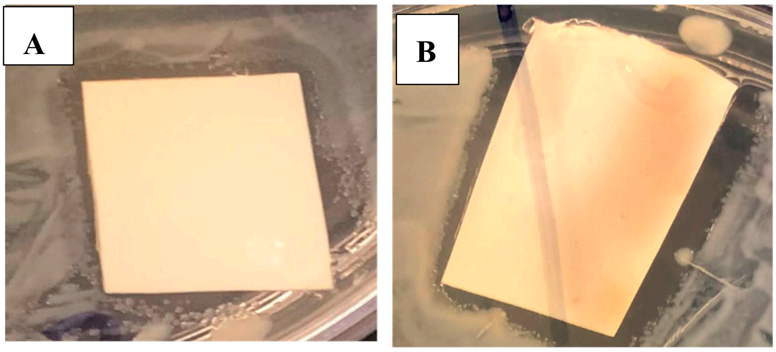

Fig S3. Anti-bacteria effect on *E. coli* with membrane without membrane (A) and embedded with silver (B).
